# Autologous micrografting improves regeneration of tissue-engineered urinary conduits in vivo

**DOI:** 10.1038/s41598-024-72876-0

**Published:** 2024-09-25

**Authors:** Nikolai Juul, Mahboobeh Amoushahi, Oliver Willacy, Micki Ji, Chiara Villa, Fatemeh Ajalloueian, Clara Chamorro, Magdalena Fossum

**Affiliations:** 1 Laboratory of Tissue Engineering, Faculty of Health and Medical Sciences, Rigshospitalet, University of Copenhagen, Copenhagen, Denmark; 2 Division of Pediatric Surgery, Department of Surgery and Transplantation, Rigshospitalet Copenhagen University Hospital, Copenhagen, Denmark; 3 Department of Forensic Medicine, University of Copenhagen, Copenhagen, Denmark; 4 Department of Health Technology, Technical University of Denmark, Kgs. Lyngby, Denmark; 5 Laboratory of Tissue Engineering, Department of Women’s and Children’s Health, Karolinska Institutet, Stockholm, Sweden

**Keywords:** Tissue Engineering, Guided tissue regeneration, Micrograft, Miniature swine, Congenital abnormalities, Pediatrics, Urology, Urogenital models, Paediatric urology, Biomedical engineering

## Abstract

Urogenital reconstructive malformation surgery is sometimes hampered by lack of tissue for the repair. We have previously shown that autologous micrografting allows for single-staged scaffold cellularization after surgical implantation. Here, a collagen-based scaffold reinforced with biodegradable mesh and a stent was implanted as a bladder conduit in ten full-grown female minipigs. We aimed to assess short-term regenerative outcomes, safety, and feasibility of implanting tubular urinary micrografted scaffolds versus acellular controls. Five scaffolds were embedded with autologous urothelial micrografts harvested perioperatively. After six weeks, all animals were assessed by cystoscopy, CT-urography, and microanatomical assessment of the urinary conduits. The procedure proved technically feasible within the confines of a regular surgical theater, with duration-times comparable to corresponding conventional procedures. No animals experienced postoperative complications, and all implanted conduits were patent at follow-up. Improved tissue regeneration was observed in the micrografted conduits compared with the acellular controls, including increased luminal epithelialization, increased cell proliferation, decreased cell apoptosis, and increased conduit vascularization. We concluded that single-staged on-site construction and implantation of tissue engineered urinary conduits proved feasible and safe, with improved regenerative potentials in micrografted conduits. This study presents a new approach to urinary conduits, and merits further investigations for advancement towards clinical translation.

**Supplementary Information:**

The online version contains supplementary material available at 10.1038/s41598-024-72876-0.

## Introduction

Urinary diversion is indicated in a variety of urological conditions where proper emptying of the bladder is hindered due to either anatomical or functional abnormalities. In patients with need for long-term catheterization, a continent channel (i.e., a conduit) is often surgically reconstructed by intestinal transposition to the abdominal skin level to spare patients of urethral catheterization. The conventional procedure for this implantation utilizes the cecal appendix which is harvested, without compromising the mesenteric blood supply, and anastomosed between the urinary bladder and a skin level stoma^1–4^. If not available, small intestine is interposed in a similar manner. However, applying gastrointestinal tissue in the reconstruction of the urinary tract often comes at the price of various complications such as mucus formation, chronic infection, stricture development, bladder stones, and even malignancy^5–9^. Whereas the appendix may have been previously removed in some patients, various alternative anatomical abnormalities make the surgery unsuitable for others. Furthermore, intraperitoneal surgery carries the risk of postoperative abdominal adhesions and subsequent complications. In recent years, several tissue-engineered solutions have tried to minimize these complications by means of various biocompatible tubular scaffolds designed for surgical implantation^10–18^. The variety of previously attempted approaches for the construction of a tissue-engineered scaffold to suit this purpose encompass an array constructs. Commonly, a biological or synthetic scaffold is applied as the structural backbone, and this in term can be cellularized with different cell-types and enriched with various growth factors or other pro-regenerative substanses^10,19,20^. Biological scaffolds are often made from decellularized organ tissue of varying origin and with high biocompatibility, however, deviating manufacturing protocols make them susceptible to limited mechanical integrity and stability^11,21,22^. Whereas synthetic scaffolds excel in their high reproducibility and potential for mass-scale distribution, the biodegraded metabolites of these scaffolds can give rise to unexpected adverse immunological host-reactions^23,24^. Finally, collagen hydrogels have been vastly applied in tissue engineering as a biological substrate for scaffold construction due to high biocompatibility and as a viable tissue adhesive. Nevertheless, the poor biomechanical support often necessitates and additional supportive biomaterial in a hybrid construct^24–27^. Furthermore, scaffolds are often cellularized in vitro before implantation since this process has been proposed to reduce post-implant scar tissue fibrosis and promote guided wound healing towards the in vivo establishment of physiological target-organ resemblance^28–31^. The production of these scaffolds is usually cumbersome, involving laboratory cell expansion by cell culturing, and the clinical translation has often been impeded by safety issues related to ex vivo tissue handling, a need for resource-intensive facilities, and lack of required expertise.

Here, we present a tubular scaffold, designed as a urinary conduit, which can be constructed from commonly used surgical materials, assembled and implanted with autologous tissue within the confines of a regular surgical theater, as a single-staged surgical procedure. In brief, the conduit consisted of a mesh-reinforced collagen-based scaffold seeded with autologous micrografts (i.e., dissected autologous bladder mucosa particles) which were harvested directly prior to scaffold construction. After plastic compression (expulsion of water), the scaffold was finally tubularized around a biodegradable stent and anastomosed to the anterior bladder wall. In this study, we inserted the tubular scaffold in a minipig model with six weeks observational follow-up. Our primary aims were to evaluate (1) the technical feasibility of the procedure, and (2) the safety and biocompatibility in vivo. The advantages and drawbacks of cellularized scaffolds in clinical settings have been debated, and in larger grafting areas (> 1 cm^2^) it has been proposed that acellular scaffolds are more prone to scar tissue formation, consequently resulting in stricturization and clinical stenosis^28,29^.

**Fig. 1:**
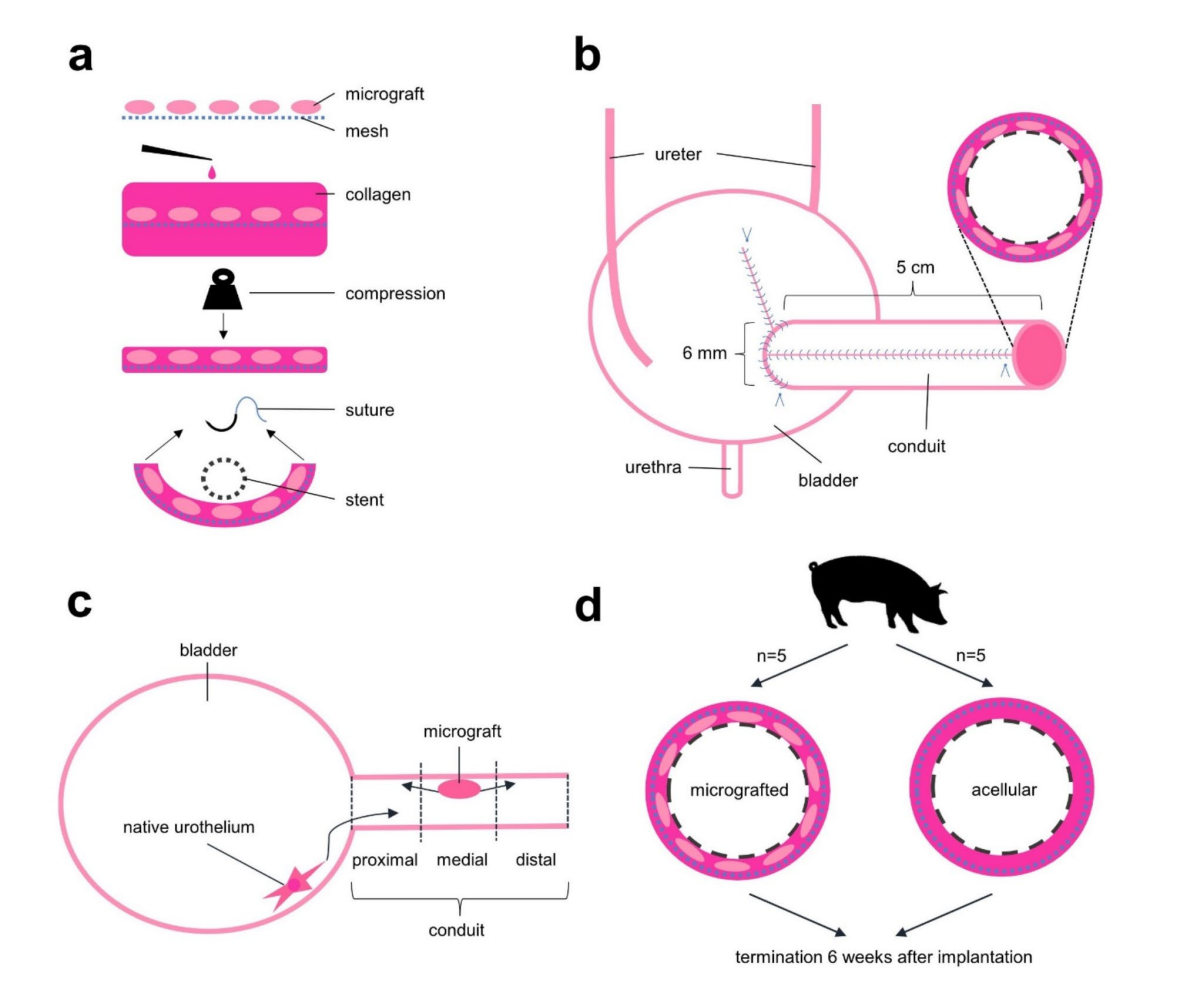
Schematic illustration of the experimental model. (a) Autologous bladder mucosal micrografts were harvested and seeded directly on a surgical polyglactin mesh. The mesh was embedded in liquid rat-tail collagen I and compressed to expel water. The scaffold was sutured around a biodegradable stent, forming the final conduit. (b) The conduit was implanted on the anterior bladder wall, at the same single-staged procedure, thereby communicating with the bladder lumen, but ligated distally. (c) The proximal, medial, and distal sections of the conduits were analyzed separately to account for native urothelium migrations. (d) To evaluate the regenerative impact of embedded micrografts, five animals with acellular conduits were included for comparison. All ten included animals were observed for six weeks before termination.

Since we expected a migration of native bladder cells proximally from the bladder anastomosis into the conduits, we aimed lastly to 3) compare the microanatomical regeneration in different sections of micrografted conduits (from proximal to distal) versus acellular, but with otherwise identical, conduits in a parallel group of control animals (Fig. 1).

## Results

### Surgical outcomes

Ten animals were implanted with the tubular scaffolds (i.e., conduits) during a mean surgical time of 2.41 ± 0.5 h (mean ± SD), including perioperative scaffold construction (mean time 31 ± 5 min). The surgical times for animals with acellular scaffolds were on average 4.2 min faster than for animals with micrografted scaffolds. Within a few hours after surgery, all animals were ambulated, urinating, and altogether regaining natural behavior. At the first postoperative day, one animal (micrografted group) developed a subcutaneous seroma cranially to the cicatrice, which had completely subsided at the fourth postoperative day. Another animal was prolonged on analgesics and antibiotics for one week due to an infected hematoma at the site of the perioperatively placed ear vein catheter (acellular group). During the six-week observational period, all animals were thriving in the stables and retained their preoperative body weight.

**Fig. 2:**
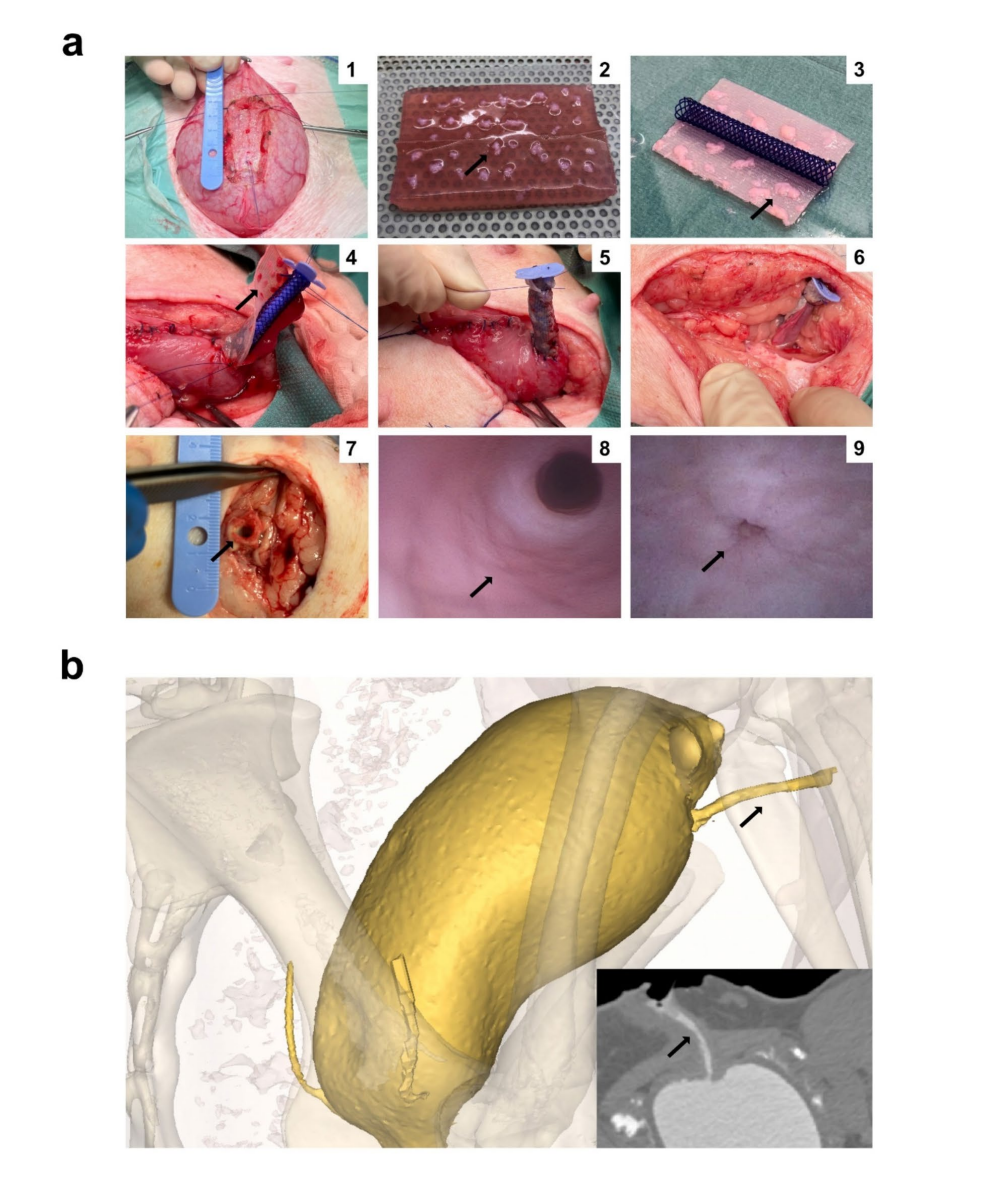
Perioperative scaffold construction and postoperative evaluations. (a) A segment of tissue was prepared for resection from the anterior bladder wall before further dissection and mincing into mucosal micrografts (1). The micrografts (marked) were embedded into the mesh-reinforced collagen (2). The compressed scaffold with adhescent micrografts (marked) before tubularization around the stent (3). Conduit implantation with luminally facing micrografts (marked) on the anterior bladder wall (4). Placement of the endoluminal ACE stopper and ligation of the distal conduit (5). Trans-fascial subcutaneous fixation of the ligated conduit (6). Post-mortem dissection of the conduit with clear urine in the lumen (marked) (7). Visible stent remnants (marked) seen with cystoscopy via the external conduit opening (8). Internal bladder anastomosis (marked) seen with cystoscopy via the native urethra (9). (b) Two- and three-dimensional representations of the bladder and conduit (marked) from post-mortem CT urography.

Post-mortem cystoscopy revealed macroscopically healthy mucosa throughout the conduit lumina in all animals. In a third animal (acellular group), a 4 mm non-obstructing bladder stone was encountered in an otherwise non-affected bladder lumen. From contrast injection CT urography, the bladder anastomosis, and all conduits, were found successfully patent and unobstructed in all animals. Tissue dissection revealed no macroscopic signs of inflammatory infiltration, necrosis, or abscess formation adjacent to the conduits in any of the animals (Fig. 2).

### Histological evaluations

**Fig. 3:**
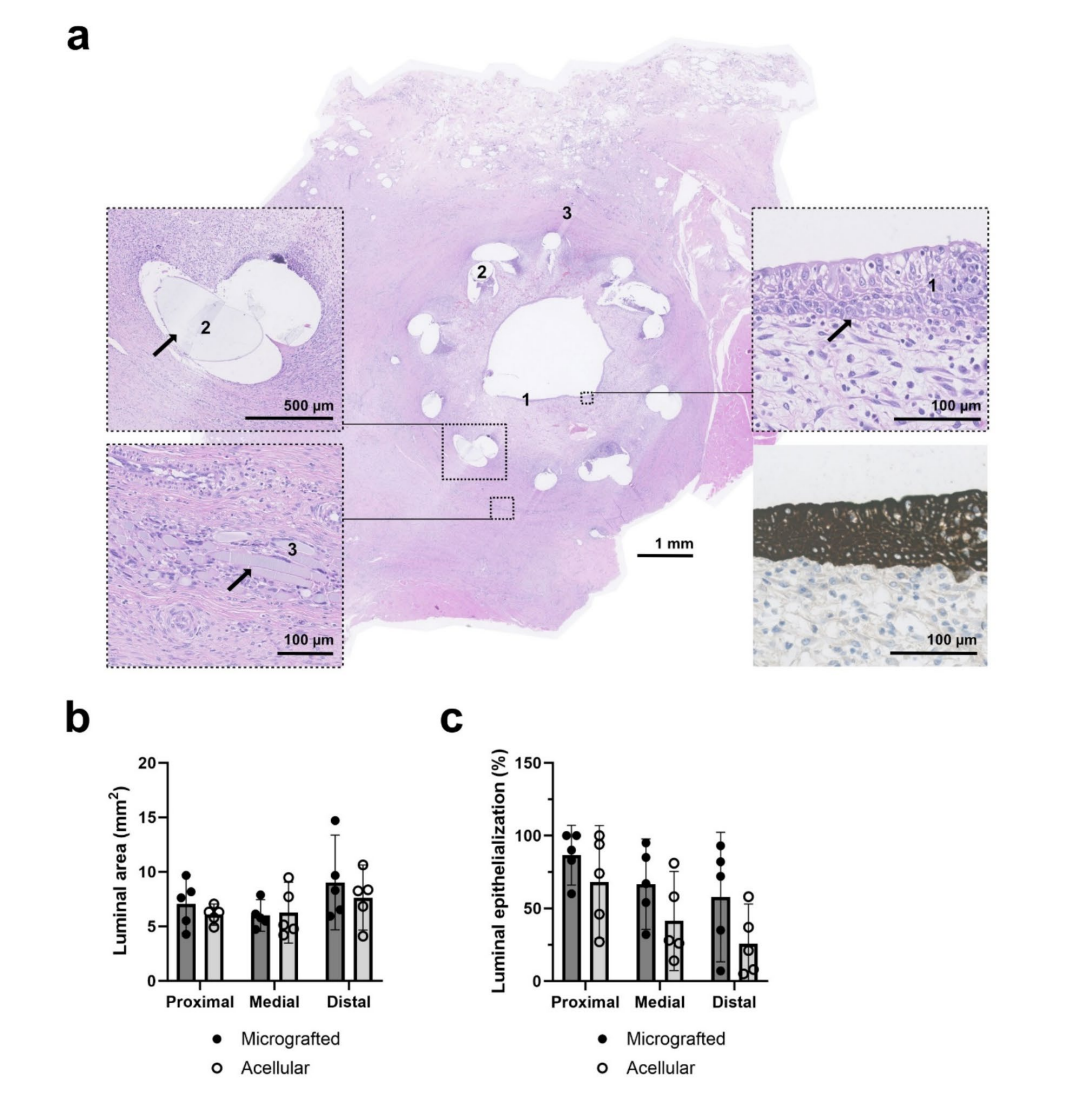
Histological evaluations of luminal area and epithelialization. (a) Distal micrografted conduit cross-sectional hematoxylin stains six weeks after implantation with marked epithelium (1), stent remnants (2), and mesh remnants (3). Cytokeratin staining was used to evaluate the circumferential degree of luminal epithelialization (bottom, right). (b) Digitally assessed luminal areas comparing the two interventional groups separately in each of the three conduit sections (means with 95% CIs). (c) Digitally assessed luminal epithelialization comparing the two interventional groups separately in each of the three conduit sections (means with 95% CIs).

The resected conduits were evaluated from histologically fixed orthogonal cross-sections throughout the conduit lumina, and, from hematoxylin stains, fragmented remnants of both mesh and stent material were still partly visible after six weeks (Fig. 3a). The cross-sectioned luminal conduit areas were digitally estimated in both groups and compared separately within each of the anatomical conduit sections (proximal to distal). Across all specimens, we observed a non-significant tendency towards larger mean luminal areas at the distal conduit sections compared with the proximal Sect. (8.6 ± 2.4 mm^2^ vs. 6.3 ± 1.9 mm^2^, *p* = 0.0974), and, in the distal conduit sections, a non-significant tendency was observed towards larger mean lumen in the micrografted group compared with the acellular group (9.1 ± 2.9 mm^2^ vs. 7.6 ± 1.9 mm^2^, *p* = 0.4854) (Fig. 3b). From cytokeratin stains, the circumferential degree of luminal epithelialization was quantified in both groups throughout the conduit sections. Epithelial lining was noted through the entirety of lumina in both groups, although sparsely distributed in the distal segments in some specimens. Extraluminal presence of epithelial cells was noted around the stent remnants in some specimens, however, no definite cystic formations were observed. Across all specimens, a non-significant tendency of decreasing mean epithelialization in the distal conduit sections was observed (proximal 77.4 ± 24.2% vs. distal 41.8 ± 31.1%, *p* = 0.1542). Furthermore, we observed a non-significant tendency towards higher mean degree of distal epithelialization in the micrografted group compared with acellular (micrografted 86.6 ± 32.1% vs. acellular 68.2 ± 19.7%, *p* = 0.2778) (Fig. 3c).

### Immunofluorescent assessments

**Fig. 4:**
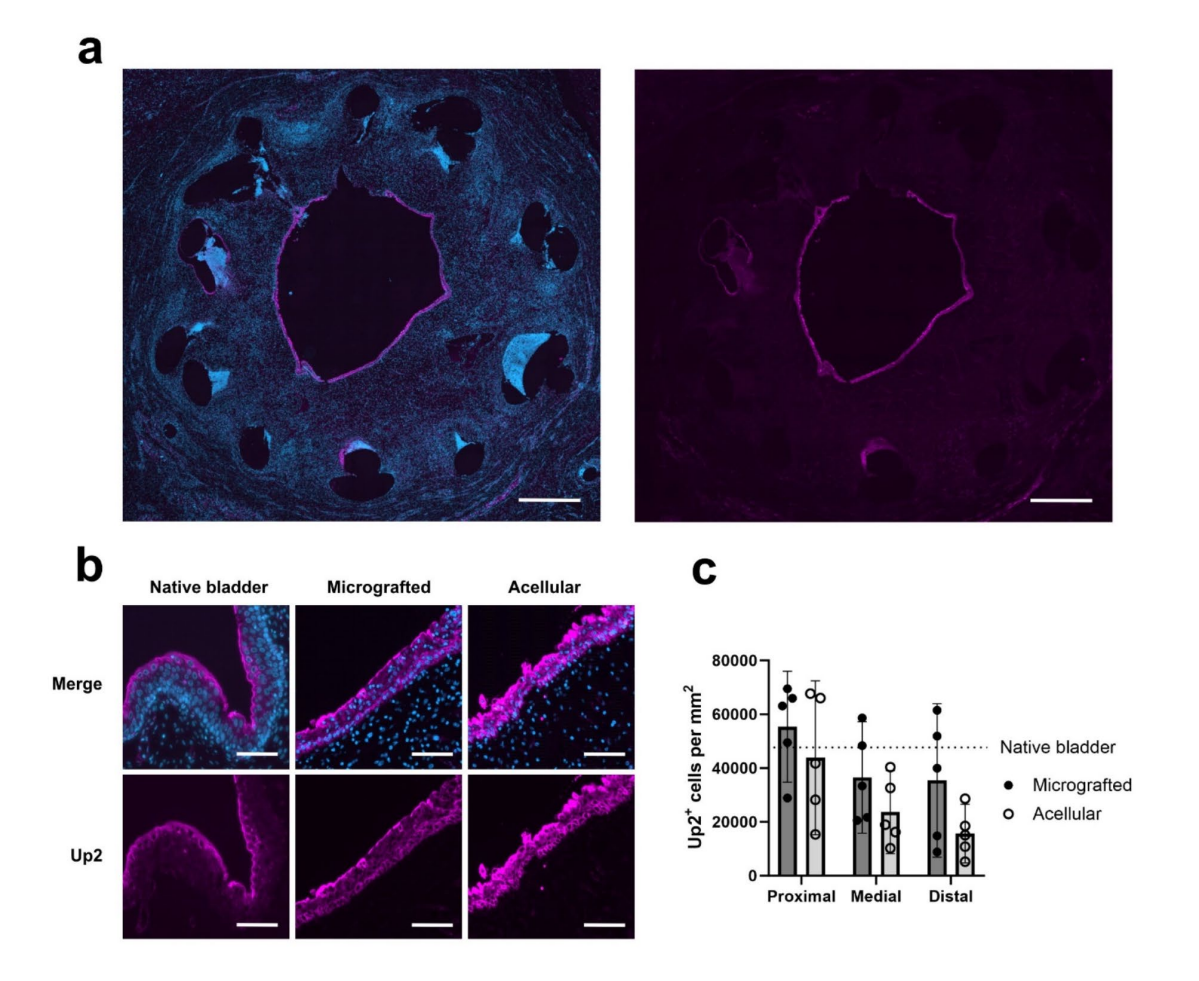
Urothelial conduit colonization. (a) Fluorescence microscopy images demonstrating the distribution of merged DAPI (blue) and uroplakin-2 (Up2, purple) positive cells (left) and Up2 alone (right) in a distal micrografted conduit section. Scale bars 1 mm. (b) Sections of merged DAPI and Up2 stains (top) and Up2 alone (bottom) in native bladder, distal micrografted-, and distal acellular conduits. Scale bars 50 μm. (c) Sectional comparisons of Up2 positive cells per mm^2^ between micrografted and acellular conduits (means with 95% CIs). Mean level of Up2 positive cells per mm^2^ for native bladder marked with dotted line.

To distinguish the degree of urothelial conduit epithelialization further, uroplakin-2 positive cells were compared between the two groups (Fig. 4a). Across all specimens, we observed a non-significant tendency of increased mean levels of urothelial cells in proximal conduit sections compared with distal sections (proximal 49,611 ± 18,873 positive cells per mm^2^ vs. distal 25,541 ± 18,405, *p* = 0.1348) and resembling levels of the native bladder wall (48,106 ± 15,169 positive cells per mm^2^). In the distal sections, more urothelial cells were found in the micrografted group compared with the acellular sections, however, not statistically significant (micrografted 35,413 ± 20,517 positive cells per mm^2^ vs. acellular 15,668 ± 7,851, *p* = 0.1099) (Fig. 4b-c).

**Fig. 5:**
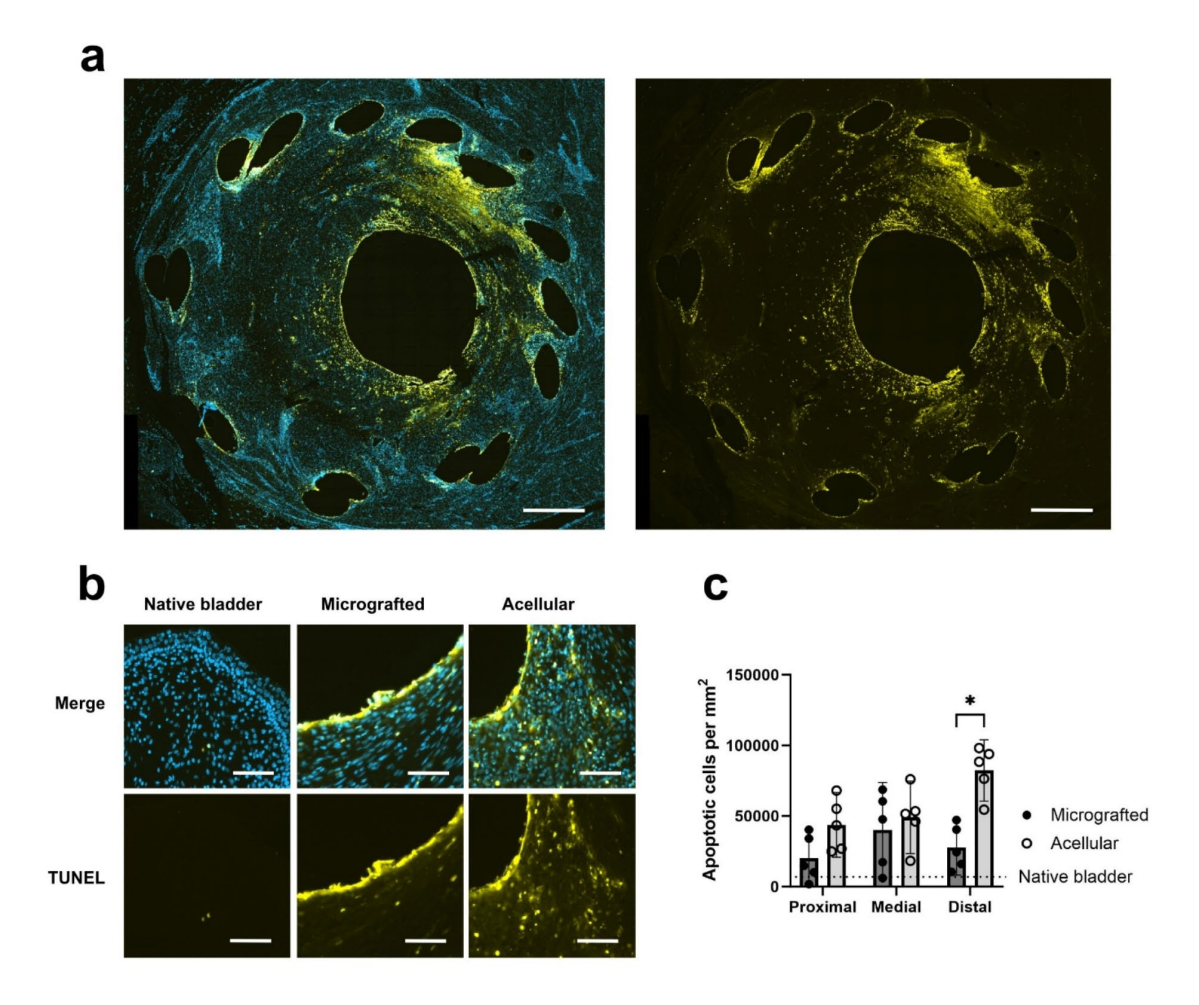
Apoptotic conduit activity. (a) Fluorescence microscopy images demonstrating the distribution of merged DAPI (blue) and TUNEL (apoptotic, yellow) positive cells (left) and TUNEL alone (right) in a distal acellular conduit section. Scale bars 1 mm. (b) Sections of merged DAPI and TUNEL stains (top) and TUNEL alone (bottom) in native bladder, distal micrografted-, and distal acellular conduits. Scale bars 50 μm. (c) Sectional comparisons of TUNEL positive cells per mm^2^ between micrografted and acellular conduits (means with 95% CIs, * for *p* < 0.05). Mean level of TUNEL positive cells per mm^2^ for native bladder marked with dotted line.

The levels of apoptotic activity throughout the conduit sections were assessed using the TUNEL assay and compared between the two groups (Fig. 5a). Apoptotic cells were predominantly present around the stent areas and at the luminal surface. Across all specimens, increased mean levels of apoptotic cells were observed in the distal conduit sections, although not statistically significant (distal 55,013 ± 31,121 apoptotic cells per mm^2^ vs. proximal 31,970 ± 19,523, *p* = 0.0762), and, as expected, the levels of apoptotic cells in all sections greatly exceeded those of the native bladder wall (3,672 ± 3,582 apoptotic cells per mm^2^). In the distal conduit sections, we observed a significant difference with more apoptotic cells in the acellular group compared with the micrografted group (acellular 82,339 ± 15,687 positive cells per mm^2^ vs. micrografted 27,687 ± 14,057, *p* = 0.0008) (Fig. 5b-c).

**Fig. 6:**
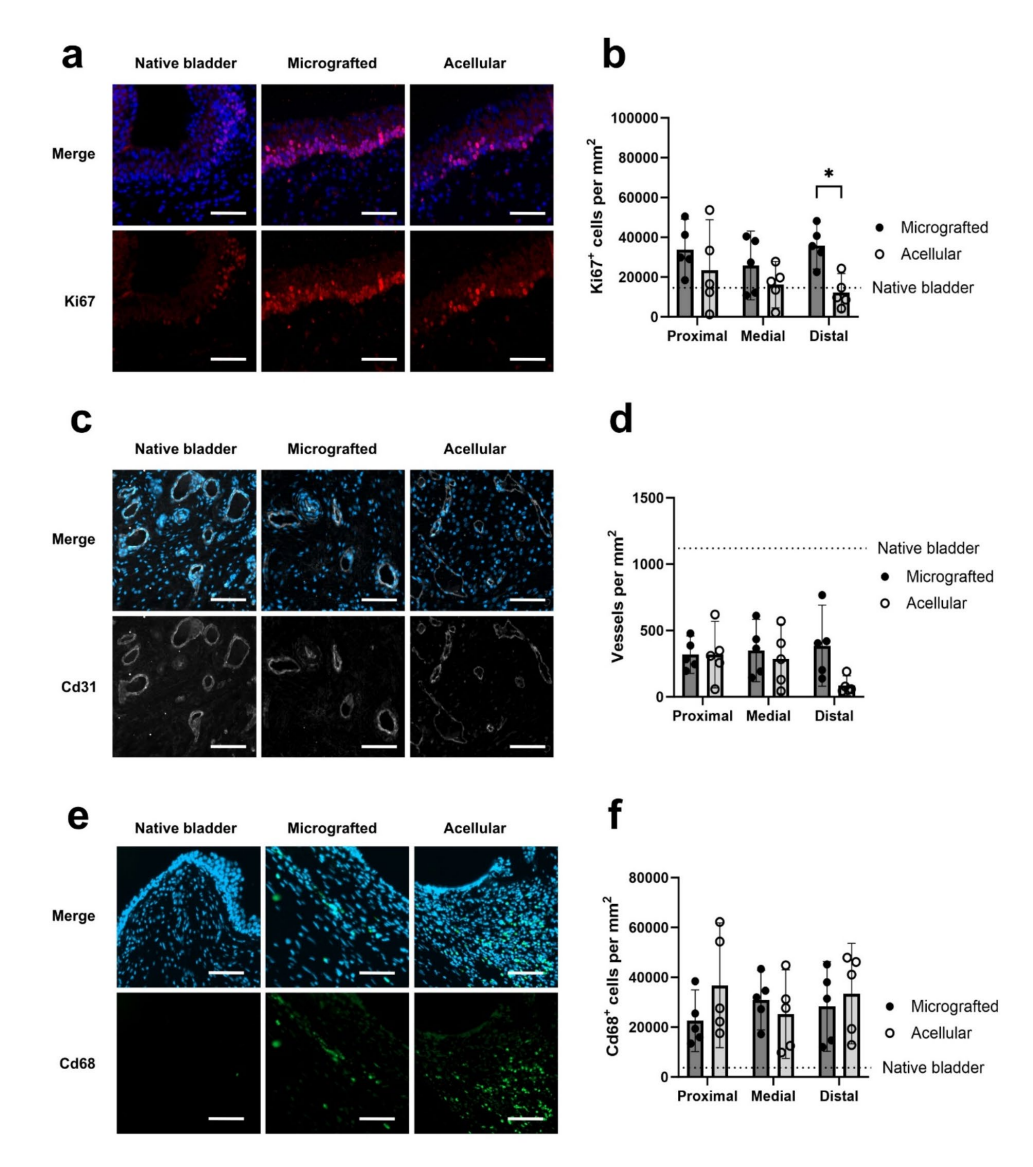
Regenerative conduit assessments. (a) Sections of merged DAPI (blue) and Ki67 (red) stains (top) and Ki67 alone (bottom) in native bladder, distal micrografted-, and distal acellular conduits. (b) Sectional comparisons of Ki67 positive cells per mm^2^ between micrografted and acellular conduits. (c) Sections of merged DAPI (blue) and Cd31 (white) stains (top) and Cd31 alone (bottom) in native bladder, distal micrografted-, and distal acellular conduits. (d) Sectional comparisons of Cd31 positive vessels per mm^2^ between micrografted and acellular conduits. (e) Sections of merged DAPI (blue) and Cd68 (green) stains (top) and Cd68 alone (bottom) in native bladder, distal micrografted-, and distal acellular conduits. (f) Sectional comparisons of Cd68 positive cells per mm^2^ between micrografted and acellular conduits. Numbers are presented in means with 95% CIs, * for *p* < 0.05. Dotted lines represent mean levels per mm^2^ in native bladder tissue. All scale bars are 50 μm.

The degree of proliferative activity throughout the conduit sections was assessed by comparing Ki67 positive cells between the two groups. Proliferative cells were predominantly seen in the epithelial areas, and, generally, the mean levels of proliferative cells across all sections slightly exceeded those of the native bladder wall (conduit total mean 24,594 ± 13,906 positive cells per mm^2^ vs. native 14,005 ± 8,407, *p* = 0.0953). In the distal conduit sections, we observed significantly more proliferative cells in the micrografted group compared with acellular (micrograft 35,915 ± 8,530 positive cells per mm^2^ vs. acellular 12,320 ± 6,843, *p* = 0.0026) (Fig. 6a-b).

To compare the level of conduit vascularization throughout the sections, Cd31 positive vascular structures were digitally quantified in both groups. Vessels were evenly distributed throughout the conduit areas, and, generally, the conduits were less vascularized than the native bladder wall (conduit total mean 291 ± 183 positive vessels per mm^2^ vs. native 1,217 ± 623, *p* = 0.0227). In the distal conduit sections, we observed a tendency towards more vessels in the micrografted group compared with acellular (micrograft mean 386 ± 219 positive vessels per mm^2^ vs. acellular 85 ± 54, *p* = 0.1289) (Fig. 6c-d).

The degree of macrophage activity throughout the conduit sections was assessed by comparing Cd68 positive cells between the two groups. Macrophages were predominantly present around the stent and mesh remnants, and, as expected, the levels of macrophages in all sections greatly exceeded those of the native bladder wall (conduit total mean 29,535 ± 13,730 positive cells per mm^2^ vs. native 3,901 ± 1,994, *p* < 0.0001). Generally, no notable differences were observed throughout the sectioned conduit or when comparing the micrografted and acellular groups (Fig. 6e-f).

## Discussion

The current surgical options for urinary diversion are manifold and morbidity in these patients remains high, altogether attesting to the issue that an optimal treatment strategy has not yet been established. Tissue-engineered solutions have long been proposed as relevant alternatives, however, the rate of clinically translated techniques remain scarce, possibly due to high complexity, limiting reproducibility, as wells as advanced and costly productions, limiting availability.

As an alternative to many of the conventional approaches in tissue engineering - often involving ex vivo cell culture and scaffold seeding - our group has tried to meet the challenges of clinical translation by circumventing some of the conventional steps in scaffold construction; small quantities of autologous tissue can be expanded in a mesh-reinforced collagen hydrogel, as mechanically minced micrografts, which can be molded and surgically handled perioperatively at the time of tissue harvest. By reimplanting the micrografted scaffold directly, and thereby leaving the final steps of scaffold reorganization and regeneration to take place within the body, we can construct and implant our scaffold as a single-staged surgery. Our overall aim is to propose a simple and accessible technique, which could allow researchers and clinicians to advance the use of tissue-engineered implants in reconstructive surgery via distinctly reproducible means. We are exploring several possible surgical indications for the technique, predominantly in hollow-organ reconstruction, with a focus on diseases within the urogenital organs. However, the basic composition of the scaffold allows for modifications to suit the specific surgical purpose – both in the choice of mesh or other reinforcing biomaterials, and via enrichment with organ-specific growth factors, antibiotics, or immunosuppressants in the collagen gel.

In this study, we evaluated the safety and feasibility of applying a mesh-reinforced, collagen-based scaffold for constructing urinary conduits in a minipig model. The scaffold was assembled and implanted perioperatively, as a single-staged procedure. Furthermore, we assessed the impact of embedding autologous urothelial micrografts into the conduits perioperatively and compared the microanatomical regeneration with acellular conduit controls. In our experience, the procedure was both technically feasible and clinically safe in a porcine animal model during a six-week follow-up. As a cornerstone in our methodology, we employed perioperative autologous micrografting as a tissue-source for scaffold cellularization, hereby aiming to circumvent the need ex vivo scaffold culture. When comparing micrografted and acellular conduits, we observed increased epithelialization and vascularization, and significantly increased levels of cell proliferation and decreased levels of apoptosis in the distal conduit segments of the micrografted animals compared with the control group. Our findings support the conclusions of previous studies, that acellular scaffolds indeed perform inferiorly to cellularized scaffolds^28,29,32^.

Since we are still in the process of assessing functionality and durability of our construct, the decision to focus specifically on urinary diversion relies partly on the relatively static biomechanical and physiological conditions of this model, which were considered suitable for meeting the primary aims of the study. In prior in vitro studies, we evaluated the tissue-laden scaffolds to justify its application in animal models. By using in vitro assays, we previously demonstrated how paracrine communications from urothelial micrografts, via extracellular vesicles, activates specific intracellular pathways related to wound healing^33^. The collagen scaffold seems to act not only as a feasible bio-adhesive, firmly fixating the micrografts to the scaffold during surgical handling, but also as a viable regenerative platform for the cells post-implantation^23,34^. We have previously been successful in implanting the tissue-laden scaffold in rodents and rabbits^32,35^, however, this large-animal study benchmarks another advancement towards human translation. Furthermore, in a pilot series evaluating the construct both in vitro and in vivo, we have previously demonstrated that, at the time of surgical implantation, the scaffold is biomechanically more robust than native bladder tissue, signifying the surgical applicability and support during take of transplant. However, as healing progresses, the implanted scaffold rapidly degrade as tissue regenerates reorganizes and produces extracellular matrix^36^. Finally, our previous in vitro assessments demonstrated how the scaffold barrier function is established as cells proliferate and expand into the scaffold^35,36^. In combination, our previous findings substantiate the feasibility of implanting the scaffold in humans, and even, potentially, in pediatric patients still undergoing growth.

Limitations of this study includes the small number of subjects included, and that the explorative nature of our study disabled us from predefining any sample size estimations, as expected complications were challenging to foresee. Despite the small study size, tendencies clearly indicated that micrografting positively affected regenerative outcomes, and, whereas less notable in the conduit sections directly adjacent to the bladder, micrografting significantly improved the tissue regeneration of more distal segments. Although our findings support the use of cellularized scaffolds, we were unable to demonstrate any difference in the rate of luminal stricturization, which is a pertinent risk in clinical settings. We interpret that the indifference between the two interventional groups was not related to inadequate micrograft effects but rather to the mechanical impacts from the implanted ACE stopper. As seen in the apoptosis assay, cells in contact with foreign body materials exhibited apoptosis markers more frequently, which could be an effect of mechanical stress post-implantation. Therefore, a longer study period, with removal of the centrally placed ACE-stopper, would further elucidate the need for micrografts to prevent stricture formation. Another obvious limitation to our study was that the conduit was not anastomosed to the skin level, but rather ligated distally, as a bladder diverticulum. A skin-level stoma would, for practical reasons, have required a much more extensive post-operative course regarding husbandry and animal surveillance, and presumably increased animal morbidity distorting the current aims.

Other limitations to the technique relate to the large group of patients requiring urinary diversion after cancer-related radical cystectomy. In our view, these patients are still not eligible for this technique since it would require an additional cell screening-mechanism avoiding the reintroduction of malignant autologous cells into the implanted scaffolds^20^. Furthermore, whereas this procedure may represent far less surgical trauma, compared with several current strategies, some patients may nevertheless still benefit from more conservative approaches (e.g., pharmacological treatment, botox injections, vesicostomy button etc.). Finally, another aspect is the need to select patients that adhere to the follow-up regimen related to the set-up of a clinical trial and long-term follow-up.

In conclusion, our study highlighted the potential of an accessible tissue-engineering methodology, with low-cost expenditure, which distinguishes itself from previous approaches by minimizing ex vivo handling of autologous tissues for the surgical repair. This unfolds new technical possibilities in diverse conditions requiring reconstructive surgical repair, and with good reproducibility qua the limited components comprising the final construct. Another advantage to this specific procedure, as opposed to utilizing the appendix or other gastrointestinal segments, is that it will allow the surgeon to implant a urinary conduit in the human pre-peritoneal space, hereby avoiding any intraperitoneal dissection with subsequent peritoneal adhesions and risk of complications (e.g., bowel obstruction, reduced fertility etc.). Importantly, the technique required only a single-staged surgical intervention, sparing the patient of subsequent anesthesia and surgical trauma - factors that have been some of the main hurdles, holding back previous tissue engineering techniques from clinical translation in reconstructive surgery. Due to the non-complex design of the tissue-laden scaffold, we are exploring important perspectives for future clinical application, however, clinical translation still requires further studies and warrants caution when selecting patients with the right clinical indication.

## Methods

### Surgical procedure

Ten full-grown female Göttingen minipigs (average one year of age, weighing 35–45 kg) (Ellegaard Göttingen Minipigs, Dalmose, DK) were included consecutively for surgery and randomized for one of the interventional groups (i.e., micrografted or acellular conduits) using an online tool (https://random.org). Sedation was induced with Zoletil^®^ (3.6 mg, Virbac, Kolding, DK) and ketamine (125 mg), and visual-guided endotracheal intubation was performed. Bilateral ear-vein catheters were installed and anesthesia was sustained with propofol (10 mg/kg/hr) and fentanyl (10 µg/kg/hr). A urinary catheter was inserted, and the bladder was filled with 250 mL of physiologically temperate isotonic saline. The animals were placed in the supine position, and the abdomen was razed and scrubbed. After two further rounds of skin cleaning with 70% ethanol, the surgical field was framed with sterile draping. A lower midline laparotomy was performed, and the intraperitoneally placed urinary bladder was mobilized to the wound. After prophylactic hemostasis on the anterior bladder wall, a 2 × 5 cm full-wall segment was excised. A proximal opening of 1 cm^2^ was left, while the remaining bladder wall was closed with a Vicryl™ 2 − 0 running suture (Ethicon, Johnson & Johnson, New Brunswick, US). Meanwhile, the mucosal layer of the resected specimen was carefully dissected, and a 2 cm^2^ mucosal was minced into 1 mm^2^ micrografts before scaffold embedding (please see separate section below). After completion of the scaffold, the final conduit was anastomosed to the remaining opening on anterior bladder wall with a PDS™ 5 − 0 running suture (Ethicon). A peritoneal flap from the pubovesical ligament was used to patch the conduit, and an intraluminal 14fr ACE stopper (Aquaflush^®^, Abena, Taastrup, DK) was centrally placed. The distal conduit end was ligated with a PDS™ 5 − 0 ligature, and saline was injected via the bladder catheter to fill the bladder and confirm patency. A trans-fascial channel was created, and the conduit was placed in a subcutaneous pocket with Prolene™ 3 − 0 sutures (Ethicon) that fixated the distal conduit and marked its placement by sutures at the skin-level location (Fig. 2a). The anterior fascia of the abdominal muscle was closed with a PDS™ 2 − 0 running suture, the subcutis was adapted with Vicryl™ 4 − 0 interrupted sutures, and the skin was closed with Prolene™ 3 − 0 running suture. After anesthetic discontinuation, the animals were extubated and observed in the stables until fully ambulant and safely able to drink and eat.

### Scaffold construction

Prior to the surgery, a solution of rat-tail collagen type I (2.06 mg/ml protein in 0.6% acetic acid, First Link Ltd, Wolverhampton, UK) was prepared as previously described^25^. Briefly, the solution was added with 10x MEM (Gibco, Thermo Fisher Scientific, Waltham, US) and pH was carefully titrated to 7.4 with 1 M NaOH (Gibco) while kept on ice, and then added with 1x MEM (Gibco) for a final collagen concentration of 1.64 mg/mL. Furthermore, a sterilized custom-made steel mold of 1 × 3 × 6 cm (height x width x length) was prepared, with an indwelling Vicryl™ fitted mesh (2 × 6 cm, cat#VM1208, Ethicon). After surgical resection and mincing, the mucosal particles (i.e., micrografts) were then manually expanded with forceps onto the mesh at a 1:6 expansion ratio (i.e., a 2 cm^2^ mucosal area was minced and evenly distributed onto the 12 cm^2^ mesh). Next, 20 mL of the collagen solution was gently poured onto the micrografted mesh (inside the sterile steel mold), and the entire construct was then transferred to a 37 °C incubator and left to solidify for five minutes. After sufficient gelification, the hydrogel was placed on a nylon mesh and the mold was removed. The hydrogel was then compressed with a weight of approximately 250 g for five minutes. After expelling water from the scaffold, the flattened scaffold was rolled around a custom biodegradable polydioxanone (PDS) stent (SX-ELLA Degradable Biliary DV stent, ELLA-CS, Trebes, CZ) measuring 5 × 0.6 cm (length x diameter) and sutured longitudinally with a PDS™ 5 − 0 running suture. The mesh was placed with the course fiber direction in the longitudinal orientation of the stent. The completed conduit was then ready for surgical implantation, as described above (Fig. 2a). For acellular conduits, scaffolds were produced and implanted as described above but without adding autologous micrografts.

### Postoperative observations

Postoperative analgesia and antibiotic prophylaxis were managed with buprenorphine (0.1 mg/kg/8hr intravenously) for the first three days, meloxicam (0.4 mg/kg/day orally) for the first four days, and trimethoprim (96 mg/day orally) and sulfadoxin (480 mg/day orally) for the first five days.

The animals were single housed during the entire postoperative period to avoid conflicts and nibbing of external vein catheters and suture material. In accordance with European legislation, each stable had visual contact with the neighboring stables through plexiglass windows and was provided daily with fresh hay, as well as toys and water supply ad libitum. The animals were carefully monitored daily for natural behavior, eating habits, urine- and stool production, and body weight was assessed weekly. Humane endpoints included clinical signs of intraabdominal bleeding, signs of pain intractable within 6 h, infections intractable within 5 days, and postoperative weight loss > 15%. At the end of the observational period (six weeks), the animals were sedated with Zoletil^®^ and terminated with a lethal pentobarbital injection (100 mg/kg intravenously).

### Postmortem assessments

After termination, the distal conduit was dissected at the skin level and the ACE stopper was removed. The urethra was closed with a plastic clamp and 250 mL of a 20:1 contrast solution of iohexol in isotonic saline (Omnipaque™ 350 mg/ml, GF Healthcare, Oslo, NO) was injected via the distal conduit opening. The animal was then assessed with a 128-slice computed tomography scanner (SOMATOM Definition A/S, Siemens, Erlangen, DE). Images were visualized using multiplanar reconstruction and all images were analyzed using the medical image processing software Materialise Mimics™ (v24.0, Materialise NV, Leuven, BE). Subsequently, an endoscopic examination of the bladder- and conduit lumina was performed with a 16.2 Fr flexcystocope (Ambu^®^ aScope™ 4 Cysto, Ambu A/S, Ballerup, DK) via the native urethra. Directly hereafter, the conduit was resected en bloc during careful evaluation of any gross anatomical findings (Fig. 2a-b).

### Histological processing

The excised specimen was fixed in 4% formalin for 24 h, and the conduit was divided orthogonally into separate equal-sized sections of either proximal, medial, and distal conduit, before further dehydration and paraffin embedding, as per standard protocol. Furthermore, full-wall bladder biopsies were resected with a 2 cm margin to the conduit anastomosis and processed in similar fashion for reference values. To assess general tissue morphology, 5 μm sections were stained with hematoxylin and eosin (CS70030-2 + CS70130-2, DAKO Agilent, US) and pancytokeratin CK-AE (Clone AE1/AE3, ID: GA053, DAKO Agilent, US) and scanned with a Visiopharm Oncotopix scanner (Hamamatsu, Shizuoka, JP) (Fig. 3a). For immunofluorescence assessments, separate sections from each conduit segment were deparaffinized and rehydrated. Antigen retrieval was performed at 95 °C for 15 min, followed by permeability enhancement using 0.5% Triton X-100 in phosphate-buffered saline (PBS) for 10 min. The slides were then applied for blocking by 10% normal donkey serum (17-000-121, Jackson ImmunoResearch Europe Ltd, Cambridge House, UK) in PBS for 30 min. Afterwards, the slides were incubated overnight at 4 °C with primary antibodies: Up2 (ab204756, Abcam, Cambridge Biomedical Campus, UK), Cd68 (sc-20060, Santa Cruz Biotechnology Inc, Bernheimer Strasse, US), Cd31 (AF3628, R&D Systems, Inc, Abingdon, UK) and Ki67 (ab15580, Abcam, Cambridge Biomedical Campus, UK), all at 1:50 dilution in PBS containing 10% donkey serum. The day after, slides were incubated in a 1:300 dilution of secondary antibodies (donkey-anti-rabbit, conjugated with Alexa Fluor 555 dye, donkey-anti mouse conjugated with Alexa Fluor 488 dye, Thermo Fisher Scientific, Waltham, US, and donkey-anti goat conjugated with Alexa Fluor 750 dye, Abcam, Cambridge Biomedical Campus, UK), and then incubated in DAPI 1 mg/ml (Chemometec A/S, California, US). Negative controls without primary antibodies were included in all conditions (supplementary Fig. 1). To reduce autofluorescence, a commercial quenching kit (Vector TrueVIEW™ Autofuorescence Quenching Kit, VECTSP-8400-15, Vector Laboratories, Burlingame, US) was use according to manufacturer recommendations, and the sample was mounted with an aqueous medium (Vectashield vibrance M, VECH-1700). In parallel, additional slides were stained with a TUNEL kit for apoptosis assay (cat#11684795910, Roche Life Sciences, Penzberg, DE), as per manufacturer instructions. Finally, the slides were applied and scanned in a Zeiss Axioscan 7 slide scanner microscope with a x20 objective (Carl Zeiss, Oberkochen, DE).

### Histological quantification

All images were analyzed using the QuPath software (v0.5.0)^37^ and blinded to the assessor (N.J.) by an external colleague before analysis. Estimation of conduit luminal areas and urothelial coverage was performed with manual annotations on H&E stains from each segment. For estimation of immunofluorescent stains, a circular region of interest was manually annotated to each image, with a standard margin of 200 μm from the outermost point marking the stent remnants. Next, a custom script for the StarDist plugin (v0.8.5) was used to estimate total number of positive cells for each stain^38^, and another custom script for the CellPose plugin (v2.2.2) was used to estimate total number vessel-like structures (based on the cd31 stain) (supplementary Table 1).

### Statistical analyses

For each anatomical conduit segment (i.e., proximal, medial, distal), the means of all biological replicates were used to compare micrografted and acellular conduits independently. Means from the native bladder biopsies were reported as positive control references for each stain. Numbers were presented as means with 95% confidence intervals (95% CIs). Continuous variables were compared using independent two-tailed t-tests, and *p* < 0.05 was considered statistically significant. Data was analyzed using GraphPad Prism (v10.1.2, GraphPad Software Inc., Boston, US).

### Ethical approvals

This experiment was carried out in an AAALAC (American Association for Accreditation of Laboratory Animal Care) accredited experimental facility in accordance with the European legislation on laboratory use of animal subjects and after ethical permission granted by the Danish Ministry of Food and Agriculture (Ref. no. 2022-15-0201-01206). The study was reported according to the ARRIVE guidelines (full checklist separately provided to the journal).

## Electronic supplementary material

Below is the link to the electronic supplementary material.

Supplementary Material 1

Supplementary Material 2

## Data Availability

Study data not presented in the publication can be made available upon request directly to the corresponding author.
