# Supplementary material for: Autologous micrografting improves regeneration of tissue-engineered urinary conduits in vivo
Source: Sci Rep. 2024 Sep 25;14:22028. doi: 10.1038/s41598-024-72876-0 (PMC11424640; doi:10.1038/s41598-024-72876-0)
Supplement: Supplementary file 1 — Supplementary Material 1 [file 41598_2024_72876_MOESM1_ESM.pdf]

Supplementary table 1. Scripts for the automated cell quantification used in the study.

Stardist classifier script

```
/**
 * This script provides a general template for cell detection using StarDist in QuPath.
 * This example assumes you have fluorescence image, which has a channel called 'DAPI'
 * showing nuclei.
 *
 * If you use this in published work, please remember to cite *both*:
 *  - the original StarDist paper (https://doi.org/10.48550/arXiv.1806.03535)
 *  - the original QuPath paper (https://doi.org/10.1038/s41598-017-17204-5)
*/

selectAnnotations()
import qupath.ext.stardist.StarDist2D
import qupath.lib.gui.dialogs.Dialogs
import qupath.lib.scripting.QP

// IMPORTANT! Replace this with the path to your StarDist model
// that takes a single channel as input (e.g. dsb2018_heavy_augment.pb)
// You can find some at https://github.com/qupath/models
// (Check credit & reuse info before downloading)
def modelPath = "C:/Users/Public/QuPath-0.4.3/extensions/dsb2018_heavy_augment.pb"

// Customize how the StarDist detection should be applied
// Here some reasonable default options are specified
def stardist = StarDist2D
    .builder(modelPath)
    .channels('DAPI')            // Extract channel called 'DAPI'
    .normalizePercentiles(1, 99) // Percentile normalization
    .threshold(0.5)              // Probability (detection) threshold
```

```
    .pixelSize(0.5)              // Resolution for detection
    .cellExpansion(5)            // Expand nuclei to approximate cell boundaries
    .measureShape()              // Add shape measurements
    .measureIntensity()          // Add cell measurements (in all compartments)
    .build()

// Define which objects will be used as the 'parents' for detection
// Use QP.getAnnotationObjects() if you want to use all annotations, rather than selected objects
def pathObjects = QP.getSelectedObjects()


// Run detection for the selected objects
def imageData = QP.getCurrentImageData()
if (pathObjects.isEmpty()) {
    QP.getLogger().error("No parent objects are selected!")
    return
}
stardist.detectObjects(imageData, pathObjects)
stardist.close() // This can help clean up & regain memory


runObjectClassifier("nikolai");
println('Done!')
```

Cellpose detection script

```
/**
 * Cellpose Detection Template script
*
 * This script is a template to detect objects using a Cellpose model from within QuPath.
 * After defining the builder, it will:
 * 1. Find all selected annotations in the current open ImageEntry
 * 2. Export the selected annotations to a temp folder that can be specified with tempDirectory()
 * 3. Run the cellpose detction using the defined model name or path
 * 4. Reimport the mask images into QuPath and create the desired objects with the selected statistics
 *
 * NOTE: that this template does not contain all options, but should help get you started
 * See all options in https://biop.github.io/qupath-extension-cellpose/qupath/ext/biop/cellpose/CellposeBuilder.html
 * and in https://cellpose.readthedocs.io/en/latest/command.html
 *
 * NOTE 2: You should change pathObjects get all annotations if you want to run for the project. By default this script will only run on the selected annotations.
 */


def server = getCurrentServer()
def cal = server.getPixelCalibration()
pixelSizeCellpose = 4*cal.getPixelWidthMicrons()
print pixelSizeCellpose


selectAnnotations()
```

```
// Specify the model name (cyto, nuc, cyto2, omni_bact or a path to your custom model as a string)
def pathModel = 'cyto2'
def cellpose = Cellpose2D.builder( pathModel )
        .pixelSize( pixelSizeCellpose )              // Resolution for detection in um
        .channels( 'AF750' )                         // Select detection channel(s)
//      .tempDirectory( new File( '/tmp' ) ) // Temporary directory to export images to. defaults to 'cellpose-temp' inside the QuPath Project
        .preprocess( ImageOps.Core.multiply(-1) )            // List of preprocessing ImageOps to run on the images before exporting them
//      .normalizePercentilesGlobal(0.1, 99.8, 10) // Convenience global percentile normalization. arguments are percentileMin, percentileMax, dowsample.
//      .tileSize(1024)                  // If your GPU can take it, make larger tiles to process fewer of them. Useful for Omnipose
//      .cellposeChannels(1,2)           // Overwrites the logic of this plugin with these two values. These will be sent directly to --chan and --chan2
//      .cellprobThreshold(0.0)          // Threshold for the mask detection, defaults to 0.0
//      .flowThreshold(0.4)              // Threshold for the flows, defaults to 0.4
       .diameter(80)                     // Median object diameter. Set to 0.0 for the `bact_omni` model or for automatic computation
//      .useOmnipose()                   // Use omnipose instead
//      .addParameter("cluster")         // Any parameter from cellpose or omnipose not available in the builder.
//      .addParameter("save_flows")      // Any parameter from cellpose or omnipose not available in the builder.
//      .addParameter("anisotropy", "3") // Any parameter from cellpose or omnipose not available in the builder.
//      .cellExpansion(5.0)              // Approximate cells based upon nucleus expansion
//      .cellConstrainScale(1.5)         // Constrain cell expansion using nucleus size
//      .classify("My Detections")       // PathClass to give newly created objects
//      .measureShape()                  // Add shape measurements
//      .measureIntensity()              // Add cell measurements (in all compartments)
//      .createAnnotations()             // Make annotations instead of detections. This ignores cellExpansion
```

```
//        .simplify(0)                    // Simplification 1.6 by default, set to 0 to get the cellpose masks as precisely as possible
        .build()

// Run detection for the selected objects
def imageData = getCurrentImageData()
def pathObjects = getSelectedObjects() // To process only selected annotations, useful while testing
// def pathObjects = getAnnotationObjects() // To process all annotations. For working in batch mode
if (pathObjects.isEmpty()) {
    Dialogs.showErrorMessage("Cellpose", "Please select a parent object!")
    return
}

cellpose.detectObjects(imageData, pathObjects)

// You could do some post-processing here, e.g. to remove objects that are too small, but it is usually better to
// do this in a separate script so you can see the results before deleting anything.

println 'Cellpose detection script done'

import qupath.ext.biop.cellpose.Cellpose2D
```
