# Supplementary figures and images for: Autologous micrografting improves regeneration of tissue-engineered urinary conduits in vivo
Source: Sci Rep. 2024 Sep 25;14:22028. doi: 10.1038/s41598-024-72876-0 (PMC11424640; doi:10.1038/s41598-024-72876-0)

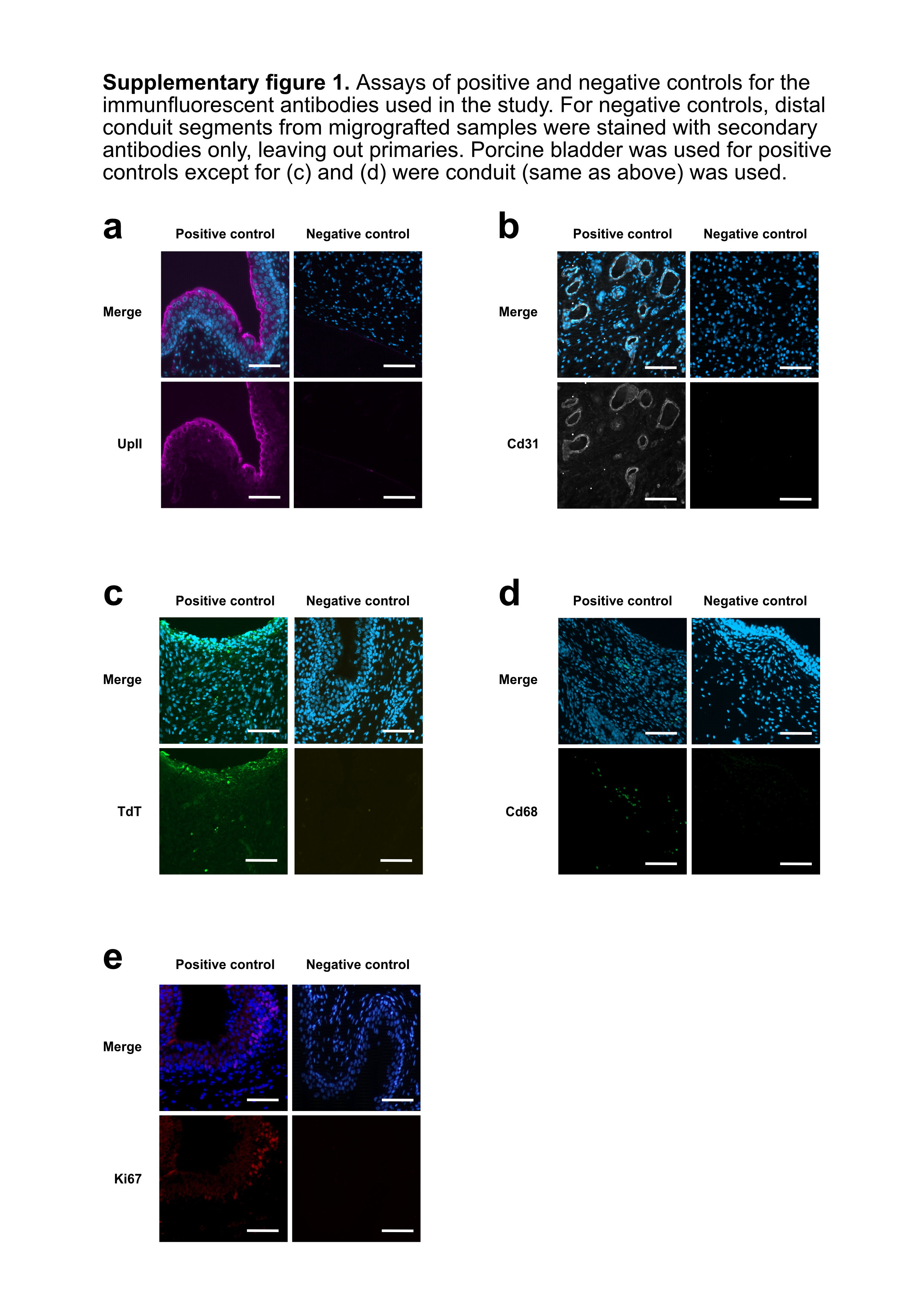

Supplement: Supplementary file 2 — Supplementary Material 2 [file 41598_2024_72876_MOESM2_ESM.jpg]
